# Optimal water resource allocation considering virtual water trade in the Yellow River Basin

**DOI:** 10.1038/s41598-023-50319-6

**Published:** 2024-01-02

**Authors:** Hao Wang, Tao Ma

**Affiliations:** 1 School of Economics and Management, Harbin Institute of Technology, Harbin, 150001 China; 2 State Key Laboratory of Urban Water Resource and Environment, Harbin, 150001 China

**Keywords:** Environmental economics, Environmental impact, Sustainability

## Abstract

Water can be redistributed physically and virtually. We explored water allocation optimization to mitigate water stresses by constructing a physical–virtual dual water system and optimizing the 1987 Yellow River water allocation scheme. We calculated the virtual water volume, identified the virtual in-basin, out-of-basin, and export water volumes, and compared the total regional water demand (i.e., combined physical and virtual water volumes) with regional water planetary boundaries to optimize basin water allocation schemes. Virtual water accounted for > 90% of the total regional demands, whereas physical flows did not significantly impact them. Moreover, allocation quotas for Qinghai and Inner Mongolia should be reduced by 0.113 and 1.005 billion m^3^, respectively, for sustainability. Furthermore, improving the efficiency of water-intensive sectors and limiting virtual water outflows from heavy industry to out-of-basin sectors are vital to water intensification. Increased attention should be directed toward physical–virtual water demands than the current focus on supply-oriented water allocation.

## Introduction

Water resources are fundamental to the socio-economic development and the stability of residential life in the Yellow River Basin^1^. As the primary allocable Yellow River resource, water resource allocation is an important policy tool for basin subsystem coordination, such as economy, society, and ecology^2^. Water allocation changes in a basin can directly cause regional economic fluctuations and trigger external economic changes. However, industrial sector interactions reshape the basin water allocation patterns^3^, e.g., the Yellow River Basin^4–6^, the South-to-North Water Transfer Project^7–9^, the Pearl River Basin^10,11^, the Colorado River Basin^12,13^, etc. Therefore, methods to optimize water resource allocation in the basin and adapt regional development strategy goals to total regional water resources constraints are crucial to the sustainable development of the basin.

In 1987, the State Council promulgated the Yellow River Water Availability Allocation Plan, the first basin-wide water allocation plan in China, ending years of regional disordered water use^4^. This basis for the allocation, utilization, and protection of Yellow River water resources was implemented to coordinate the water needs of nine provinces along the Yellow River for 35 years. This scheme protects socio-economic development and residential livelihood in these coastal regions while maintaining a healthy basin ecosystem^14^.

However, with rapid socio-economic development, the optimization objectives of the Yellow River water system must be updated. The 1987 water allocation scheme was initially established to prioritize water supply for “people’s daily requirements, national key construction industries, and downstream sand flushing into the sea”. In 2019, the ecological protection and high-quality development strategy of the Yellow River proposed a water allocation target that emphasized the importance of reasonable water allocation for production, residential life, and the ecosystem^15^. Furthermore**,** the spatial heterogeneity of water endowments in the basin caused by climate change has led to a change in water supply patterns^16,17^. Climate change may affect the total allocable water of a basin in various ways, primarily through changes in temperature and precipitation and the intensification of the frequency and degree of extreme climate events, which will significantly affect the available amount and quality of water use and thus basin water allocation scheme^18^.

Agriculture is the leading sector in virtual water trade among all provinces in the Yellow River Basin^6^, and it is highly vulnerable to climatic conditions and natural disasters (such as droughts and floods)^19^. In addition to exacerbating the basin's seasonal or quality-related water scarcity, climate change will also reduce crop yields, thereby increasing virtual water trade^20^. Further, the mismatch between water allocation quotas and regional output values, combined with the increasing volume of inter-regional virtual water trade, has changed water use patterns in the basin. For example, the industrial outputs of Qinghai, Gansu, Ningxia, and Inner Mongolia Provinces are significant; however, the 1987 water allocation scheme allocated water for ecological and agricultural activities according to the upstream ecological and food security functions. The rapid development of the energy industry has increased industrial water demands in Ningxia and Inner Mongolia, which should be considered in the new water allocation scheme^21^. In addition to the virtual water system, physical water transfer projects, such as the diversions of Han to Wei and of Yellow River to Qing and Jin, have more directly changed the water supply and use patterns in the basin^22^. Finally**,** the 1987 water allocation scheme does not sufficiently consider water resource thresholds or inter-regional environmental capacity differences^23^. According to the comparison between the planned water allocation and actual water consumption of the Yellow River (1999–2017), the water consumption of four provinces, that is, Gansu, Ningxia, Inner Mongolia, and Shandong, exceeded the water allocation quota almost every year. Among them, Inner Mongolia and Shandong have the most serious excess water consumption (average multi-year excess of 935–1547 million m^3^). Moreover, the actual water consumption of Qinghai, Shanxi, Shaanxi, Henan, Hebei, and Tianjin did not meet the planned water allocation (the actual water consumption in Shanxi Province < 1/2 of the water sharing quota and that of Hebei and Tianjin Provinces < 1/3 of the quota).

Contemporary research into the optimal allocation of water resources in the basin has not sufficiently considered the economic trade flows of water resources. Optimal water allocation in the basin predominantly occurs physically via equal division for the number of regions, considering the proportion of regional economic output and population as a negotiated division^24^, or via establishing water transfer projects for water recharge^3^. Large-scale human production and trade activities have changed the cyclic processes of the physical water system in the basin^25^; however, the virtual water system, representing the socio-economic water cycle, is also an important part of this complex system^26^. Virtual water is implicit in a product and is traded between regions based on regional water production efficiency and comparative water product advantages^27^. It is cheaper than physical water; therefore, when physical water in the region cannot meet the demand, the local water supply can be supplemented by importing foreign water products.

Studies have considered both physical and virtual water systems for optimizing water resource allocation, such as applying bottom-up methods to calculate virtual water for agricultural products^28^, separately calculating virtual water systems through blue and green water^29^, or assessing the physical water system using TOPSIS method^30^ or dividing into surface water and groundwater^31^. In contrast, the multiregional input–output model provides a comprehensive industry classification as a complement, clearly showing the differences in virtual water systems across different regions and industries. However, studies have only used it for the optimal allocation of agricultural water^32^. Few researches have optimized water resource allocation considering WPBs, including both physical and virtual water subsystems^33^. This physical–virtual dual water system is a complete expression of regional water demands, and the water planetary boundary (WPB) provides scientific thresholds for allocation. The WPB specifies global water security thresholds for human development from a supply perspective. Water scarcity in the Yellow River is a regional phenomenon that requires the extrapolation of global-scale water boundaries to the basin scale. The planetary boundary framework has strict environmental boundaries, posing a significant challenge for evaluating the sustainability of different land types. Currently, no unified standard is available for identifying basin-scale WPB^34^.

This study aims to fill this gap by assessing inter-regional virtual water trade volumes through an input–output approach and optimizing water allocation based on basin WPBs, using the Yellow River as a case study. The main objectives were to evaluate the virtual water volume of an area from the basin to in-basin, out-of-basin, and export flows; analyze the total physical–virtual water in each region of the basin; compare these values to the WPB to evaluate the amount of excess or surplus water, and optimize the results of the basin physical–virtual dual water system under WPB constraints. Our results indicate differences between physical and virtual water in the basin among eight sectors and support using the WPB as a sustainable development tool with optimal water allocation methods.

## Methods

### Modeling framework

The basin physical–virtual dual water system is a complex system governed by the flow of physical water and the socio-economic components of virtual water^35^. The previous basin water sharing scheme only focused on the allocation and utilization of physical water systems, ignoring the significant coupling characteristics and mutual feedback in physical–virtual dual water systems^36^. With unceasing socio-economic development, coastal water utilization of the basin has increased dramatically. On the one hand, water resource departments consider the limited physical water resources deployed in the socio-economic system, facilitating physical water system management in the basin. On the other hand, water resources enter the local and foreign socio-economic systems through local production and inter-regional trade activities, respectively. This virtual water system portrays the reallocation of water resources accompanying the socio-economic production and exchange of regional products, and is linked to the physical water system.

**Figure 1:**
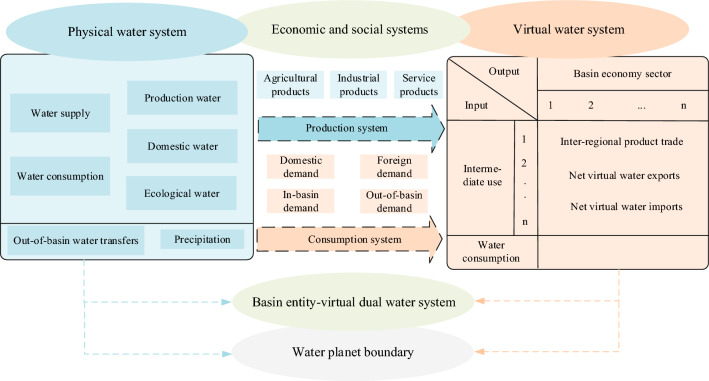
The basin physical–virtual dual water system shows each module, the items between modules, and the possible water input in each module.

The accuracy of water demand analyses can greatly affect water allocation scheme designs. Therefore**,** this study categorized the physical water system according to the incoming water mode (i.e., water transfer from outside the basin, precipitation, and water basin water resources) into production, domestic, and ecological water. These categories can further be aggregated based on the classification by use, resulting in a distinction between water supply and water consumption. Furthermore, the physical water system establishes connections with the virtual water system through the economic and social systems. In terms of production, agricultural, industrial, and service products correspond to different virtual water production systems. In terms of consumption, the virtual water system can be divided into local or foreign demand, in- or out-of-basin demand, and domestic or foreign demand according to the product flow to the consumption areas. In order to express the water demand of the basin area more thoroughly, this paper uses such indicators to establish the basin physical–virtual dual water system model, as depicted in Fig. 1.

Therefore, we obtained the excess and surplus water use of the region by comparing the total physical–virtual water demand of the region with the total water availability boundary, which was an optimization principle of the watershed water sharing scheme. Overall, this paper applies the system theory concept to establish a physical–virtual dual water system model of the basin and optimize the traditional water allocation scheme. The relationship between individual areas and the overarching basin, new development goals, and water transfer processes were managed to achieve reasonable water allocation in the basin.

### Virtual water system allocation

We used multiregional input–output models for quantitative accounting of in-basin virtual water systems. Virtual water content is dominantly measured using either the bottom-up product life cycle approach, which calculates the virtual water content from the product side^37^, or the input–output relationship between industrial sectors^38^. The use of input–output models to estimate virtual water provides a clear picture of the transfer trends of virtual water between basin areas and industries. The flow path of the virtual water system in the Yellow River Basin is shown in Table A1.

We calculated the standard structure of the multiregional input–output table for the Yellow River based on the multi-sectoral input–output table for China. Due to data availability limitations, we used a non-competitive model^39^, which assumed that products imported, transferred from outside the Yellow River, and obtained from the Yellow River differed. Therefore, these products do not have a competitive relationship as substitutes. The industry is divided into eight sectors, and the specific division criteria are detailed in Table A2. Finally, the Yellow River's 8 × 8 multiregional input–output is formed. The model is composed of 8 × n linear equations and satisfies the following relations:

1 $${x}_{i}^{r}=\sum_{s=1}^{8}\sum_{j=1}^{n}{z}_{ij}^{rs}+\sum_{s=1}^{8}{f}_{i}^{rs}+{e}_{i}^{r}-{o}_{i}^{r}$$

where, for an economy of sector *i*, the total output ${x}_{i}^{r}$ (in vector form) is produced for intermediate and final consumption; ${y}_{i}^{r}$ denotes the total economic output in sector *i*, region *r*; ${z}_{ij}^{rs}$ denotes the inputs from sector *i*, region *r*, to sector *j*, region *s*, to meet the intermediate use of the product; ${f}_{i}^{rs}$ denotes inputs from sector *i*, region *r*, to sector *i*, region *s*, to satisfy the final use in region *s*; ${e}_{i}^{r}$ denotes exports from sector *i*, region *r*, to foreign countries and ${o}_{i}^{r}$ denotes other terms in sector *i*, region *r*.

2 $${a}_{ij}^{rs}=\frac{{x}_{ij}^{rs}}{{x}_{j}^{s}}$$

3 $${A=\left[{A}^{rs}\right]=[a}_{ij}^{rs}{]}_{n\times n}$$

where ${a}_{ij}^{rs}$ is the direct consumption coefficient, which indicates the direct consumption of sector *i*, region *r*, for sector *j*, region *s*, per economic output unit. According to the Multiregional Input–Output Model (MRIO), the total economic input of each sector equals the total output of each sector; ${A}^{rs}$ denotes the matrix of direct consumption coefficients from regions *r* to *s*, and *A* denotes the matrix of direct consumption coefficients of the entire region

4 $$X=A\times Z+F+E-O$$

5 $$X=(I-A{)}^{-1}(F+E-O)$$

6 $$L=(I-A{)}^{-1}$$

where *X* denotes the column vector of aggregate economic output, *F* denotes the final demand matrix, *E* denotes the export column vector, and *L* denotes the Leontief inverse matrix.

1. Direct water use coefficient ${q}_{i}$.
  The virtual water in an industrial sector is based on the amount of water consumed by its final demand, including both direct and indirect virtual water. Direct virtual water measurements differ across industrial sectors. We use the water consumption in *i* sector (${W}_{i}$) divided by the total output of *i* sector (${X}_{i}$) to obtain ${q}_{i}$, which indicates the amount of water directly consumed by the industrial sector *i* per output unit in the production process.
  7 $${q}_{i}=\frac{{W}_{i}}{{X}_{i}}$$
  where the standard unit is m^3^/million. For *n* industrial sectors, the vector of direct virtual water content composition consumed per production value unit is $Q=({q}_{1}{q}_{2}\cdots {q}_{n})$.
2. Indirect water consumption coefficient ${j}_{i}$.
  In evaluating water resource consumption during industrial development, the actual water consumption of some industrial sectors may be larger than the direct water consumption. Although some industries have a small amount of direct water input in the production process, they simultaneously consume many other products as intermediate inputs, which each consume significant water resources during production. We considered the virtual water consumed during the production of these other products as indirect water resource consumption in industry *i*, that is, the indirect water consumption coefficient ${j}_{i}$.
  8 $${h}_{i}={q}_{i}+{j}_{i}$$
  where ${h}_{i}$ is the complete water use coefficient used to measure the complete virtual water consumption of sector *i*, which facilitates a more comprehensive calculation of the real water demand for industrial development. Multiplying the Leontief inverse matrix by the direct water use coefficient row vector *Q* for each sector yields the complete water use coefficient row vector $H=({h}_{1}{h}_{2}\cdots {h}_{n})$:
  9 $$H=Q(I-A{)}^{-1}=Q \cdot L$$
3. Virtual water content ${VW}_{i}$ in sector *i.*
  10 $${VW}_{i}={h}_{i}\times {F}_{i}$$

### Optimal allocation model of basin physical–virtual dual water system

The physical–virtual dual water system concept provides a more complete calculation of the total regional water demands. In this study, the virtual water system was added to the physical water system optimal allocation model. Overall, this model added new optimization objectives and changed the constraints as follows.

#### Objective function

1. Traditional Objective 1: Maximize the overall economic benefits of the basin.
  11 $${max}_{1}f\left(x\right)=\sum_{r=1}^{8}GDP=\sum_{r=1}^{8}\sum_{i=1}^{8}\frac{{W}_{ri}}{{h}_{ri}}$$
  The maximized economic benefits of water resource allocation in the basin were expressed by maximizing the sum of the industrial output values of eight sectors in eight provinces along the Yellow River. The complete water use coefficient ${h}_{ri}$ for sector *i*, region *r*, was calculated by Eq. (9), which expressed the amount of water consumed per 10,000 yuan of sectoral economic output. The water consumption of sector *i*, region *r*, that is, ${W}_{ri}$ was then divided by the complete water use system, and the results were added to obtain the economic value of each sector.
2. Traditional objective 2: minimize the overall pollutant emissions from the basin.
  12 $${min}_{2}f\left(x\right)=\sum_{i=1}^{8}{a}_{i}{W}_{i}=\sum_{i=1}^{8}{a}_{i}\sum_{r=1}^{8}{W}_{ri}$$
  The pollutant concentration coefficients ${a}_{i}$ for each industrial sector’s effluent discharge are obtained according to the “Announcement on Effluent Coefficients and Material Accountancy Methods for Calculating Pollutant Emissions” issued by the Ministry of Ecology and Environmental Protection in 2017. It was multiplied by the overall water consumption ${W}_{i}$ in sector i of the basin to obtain the pollutant discharge of each industrial sector.
3. Updated Objective 3: Minimize the overall water shortage in the basin.
  13 $${min}_{3}f\left(x\right)=\sum_{r=1}^{8}T{W}_{r}-{WPB}_{r}$$
  This physical–virtual dual water system calculated the total water demand $T{W}_{r}$ for region *r*. The minimization of the sum of its difference with the water boundary ${WPB}_{r}$ of each region represented the minimization of the overall water shortage in the basin.
  14 $$T{W}_{r}=P{W}_{r}{+NVWI}_{r}$$
  Water resource demands in region *r* included physical and virtual water demands; therefore, their sum provided the total water resource demands of region *r*, that is, $T{W}_{r}$.
  15 $$P{W}_{r,t}=\left\{\begin{array}{c}{W}_{r,t}^{r,t}+{W}_{r,t}^{s,t}\\ {W}_{r,t}^{Production}+{W}_{r,t}^{Domestic }+{W}_{r,t}^{Ecological}\end{array}\right.$$
  Figure 1 shows that the basin physical water system ${PW}_{r}$ can be calculated by classifying its use or function, respectively.
  16 $$NVW{I}_{r}={VW}_{s}^{r}-{VW}_{r}^{s}$$
  17 $${VW}_{r}^{s}=\sum_{i=1}^{n}{VW}_{r,i}^{s,i}$$
  The net inflow of virtual water *NVWI*_*r*_ represents the demand for water of the virtual water system in region *r*, which can be calculated from the virtual water trade volume ${VW}_{s}^{r}$ that flows from the foreign region *s* to local region *r* minus the virtual water trade volume ${VW}_{r}^{s}$ flowing from local region *r* to foreign region *s.* Whereas the virtual water trade volume of region *r* was obtained by summing the virtual water trade volume of all industrial sectors.

#### Constraints and model solution

18 $$s.t.\left\{\begin{array}{c}\begin{array}{c}\sum_{r=1}^{8}\sum_{i=1}^{8}\frac{{W}_{ri}}{{h}_{ri}}\ge min{Z}_{1}(t)\\ {W}_{r,t}^{Ecological}\ge {Z}_{2,r}\left(t\right)\\ \sum_{r=1}^{8}{W}_{r,t}^{Ecological}\ge {Z}_{3}\left(t\right)\\ {W}_{r,t}^{Domestic }\ge {Z}_{4}\left(t\right)\end{array}\\ \dots \\ \sum_{r=1}^{8}{W}_{r}\le \sum_{r=1}^{8}{WPB}_{r}\end{array}\right.$$

1. Traditional Constraint 1: constraints for optimal allocation models of physical water systems.
  The relevant constraints from the previous optimization model for the physical water system in the Yellow River were followed, as described in the reference^40^. The first constraint $min{Z}_{1}(t)$ represents the minimum planning value of the regional GDP required to be achieved according to the socio-economic development plans of the eight studied provinces. The second constraint is that the ecological water ${W}_{r,t}^{Ecological}$ in region *r* is not lower than the minimum ecological water consumption ${Z}_{2,r}(t)$. Moreover, the ecological water quantity ${W}_{t}^{Ecological}$ in the Yellow River is not lower than the overall ecological water consumption in the basin ${Z}_{3}(t)$. Finally, the last constraint ensures that water consumption by production activity does not encroach on residential domestic water use. Therefore, the optimized water allocation scheme required the regional domestic water consumption to not be reduced.
2. Updated Constraint 2: Total water resources of the basin do not exceed the water planetary boundary.
  The constraint that the total water allocated to the basin did not exceed the WPB of the basin can be expressed by Eq. (18). The WPB is a top-down translation of the total water resource constraint at the global-national-basin level, that is, it is a delineation of the basin-scale global safe operating space. This allocation process involves complex fairness issues. Additionally, among existing studies on complete biophysical boundaries, fewer studies are on WPBs, with no globally accepted applicable principles.
  First, the global water scarcity-related indicators are considered as WPB indicators. Per capita water scarcity is the most widely used indicator for assessing global water stresses, which predominantly considers domestic water use of the population, but does not sufficiently consider productive and ecological water use. Furthermore, per capita water is used as an assessment indicator of the extent of water scarcity in a region by classifying it into four categories: no stress, stress, scarcity, and absolute scarcity. Therefore, using the product of per capita water resources and population size as the regional WPB is unreasonable. However, Li et al. established two indicators to quantify the overexploitation of local and global water resources due to the demand for goods and services in Chinese provinces and cities, obtaining the WPB thresholds for Chinese provinces in terms of excess and residual water footprints^41^. Lade et al. set the WPB at 40% of the total renewable water resources of the Earth^42^.
  The Opinions of the State Council on Implementing the Strictest Water Resources Management System, released in 2012, proposed the strictest water resource management system and defined the “three red lines” of total water use, water use efficiency, and water function zone pollution limitation for each region^43^. Although water resource management in China occurs mostly at the national and provincial administrative levels, this study focused on assessing whether basin-scale water resource use exceeds the resource use thresholds for sustaining human survival and development on Earth and using this as a basis to improve the basin water allocation scheme. The total water use control indicator among these “red lines” provides an operational criterion for basin water use; therefore, the total water use control indicator is chosen to define the biophysical boundary of basin-scale water use, i.e., the WPB^44^. Considering the water resources management system in China, the WPB in the Yellow River Basin is mainly limited by the Decision on Accelerating Water Resources Reform and Development issued by the State Council of the CPC Central Committee in 2010^45^ and the Performance Assessment Method for Implementing the Strictest Water Resources Management System issued by the General Office of the State Council in 2013^46^. The policy sets “red lines” for water resource use in 31 provincial departments in China for 2015, 2020, and 2030. The WPBs specify only the present and potential future thresholds of water availability in a region, and the allocation of water resources across specific sectors can be used to coordinate the total WPBs in the basin area. These regional WPB reference values are shown in Table 1.

**Table 1:** Reference values for the Yellow River Water Planetary Boundary in 2017.

Province	Index		
	Per Capita water resources (m^3^/person)	Total water resources × 40% (billion m^3^)	Three red lines (billion m^3^)
Qinghai	433.1	23.572	3.715
Gansu	443.5	6.592	12.48
Ningxia	974.3	0.368	6.347
Inner Mongolia	744.7	21.48	19.9
Shaanxi	243.2	13.336	10.2
Shanxi	202.9	3.76	7.64
Henan	244.9	11.488	26
Shandong	210.0	6.736	29.251
3. Updated Constraint 3: According to the principle of “Big Stability, Small Adjustment” to optimize the basin water resource allocation scheme.
  The optimization principle of the 1987 Yellow River water allocation scheme follows the “Big Stability, Small Adjustment” principle proposed by the Yellow River Basin Commission. A complete “rollback” of the existing basin water scheme would be practically infeasible, and would imbalance water use patterns in coastal areas and generate a negative impact on socio-economic development and residential life. Therefore, the “Big Stability” principle implies that the ecological water quotas in the existing allocation scheme are ensured and consider the new regional water resource demands. Thus, the 1987 water allocation scheme is a benchmark to apply the “Small Adjustment” principle for optimization.
  Based on this optimization principle, this paper proposes a quantitative optimization method of “replenishing and reducing excess wate”. According to Eq. (16), the difference between the total physical–virtual water $T{W}_{r}$ and *WPB*_r_ is calculated to obtain *excess water* (*EW*), that is, the amount of regional water demand that exceeds the WPB, and *surplus water* (*SW*), that is, the amount of water remaining within the WPB.
  Excess water ($E{W}_{r}$) and surplus water ($S{W}_{r}$) can be calculated by the extent of water resource over-exploitation due to the demands for goods and services in the basin. If the total local water demand exceeds the local WPB, then the water allocation for the region should be increased. Similarly, the water quota for surplus water areas should be reduced. The total amount of surplus water is increased or decreased in the same proportion as that of the regional water share (${W}_{r}$) to the total water share (${W}_{Total}$) of the basin. The final water quota increment ${W}_{r}^{*}$ was obtained for the excess water region, as follows:
  19 $$S{W}_{r}=WP{B}_{r}-{TW}_{r}>0$$
  20 $$E{W}_{r}={TW}_{r}-WP{B}_{r}>0$$
  21 $$TSW=\sum_{r=1}^{8}S{W}_{r}\times \frac{{W}_{r}}{{W}_{Total}}$$
  22 $${W}_{r}^{*}=TEW=TSW$$
  23 $${W}_{r}^{*}=\frac{{W}_{r}}{{W}_{Total}}\times TEW=\frac{{W}_{r}}{{W}_{Total}}\times TSW$$
  where *TSW* is the total surplus water of the basin, which is first calculated as the optimized total water quota, *TEW*, that can be allocated to the excess water region. Considering the same proportional allocation of *TSW* for the basin to the excess water region, the additional *TEW* for the excess water area *r* is ${W}_{r}^{*}$, as expressed by Eq. (23).
  According to “Big Stability, Small Adjustment,” the adjustment amount $\Delta {W}_{r}$ of the optimal water allocation scheme in region *r* cannot exceed ${W}_{r}^{*}$. Additionally, the total water allocation in the basin cannot be changed, only the water allocation quota between the basin regions can be changed. This results in the following constraints:
  24 $$\Delta W_{r} \le W_{r}^{*} = \left\{ {\begin{array}{*{20}l} {\frac{{W_{r} }}{{W_{{Total}} }} \times \sum\limits_{{r = 1}}^{8} {SW_{r} } ,} & {\Delta W_{r} \ge 0\;({\text{Surplus}}\;{\text{Water}})} \\ {\frac{{W_{r} }}{{W_{{Total}} }} \times \sum\limits_{{r = 1}}^{8} {EW_{r} } ,} & {\Delta W_{r} < 0\;({\text{Excess}}\;{\text{Water}})} \\ \end{array} } \right.$$
  25 $$\sum_{r=1}^{8}\Delta {W}_{r}=0$$
4. Model solution and optimization
  Overall, the optimal allocation model of the basin physical–virtual dual water system was constructed by integrating the logical relationship between the traditional physical and virtual water systems. The model was based on the “Big Stability, Small Adjustment” principle and the new WPB constraints. The study used the Non-dominated Sorting Genetic Algorithm-II (NSGA-II) to solve the model and obtain the optimized water resource allocation results. The NSGA-II method is a multi-objective genetic algorithm proposed by Deb^47^, which can be used to solve multi-objective optimization problems. The NSGA-II method has the advantages of reducing the algorithm's complexity, fast operation speeds, and good distribution of the solution set, so the technique has become a widely used multi-objective genetic algorithm. The genetic algorithm optimization process using MATLAB is shown in Fig. A2.

## Study area and data sources

### Study area and context

The mainstream of the Yellow River, the second-longest river in China, is 5464 km long and flows through nine provinces, including Sichuan Province. Additionally, the Yellow River flows through an area that is predominantly an ecological reserve in Sichuan Province, and primarily provides ecological water to the Province^48^. Therefore, the socio-economic development of Sichuan Province is not closely linked to the water resource allocation of the Yellow River. Thus, Sichuan Province was excluded from the study area. Moreover, based on the availability of the input–output table data, provincial scales were identified as the study units. Eight provinces—Qinghai, Gansu, Ningxia, Inner Mongolia, Shaanxi, Shanxi, Henan, and Shandong—were included in the study area for optimizing the Yellow River water allocation scheme.

**Figure 2:**
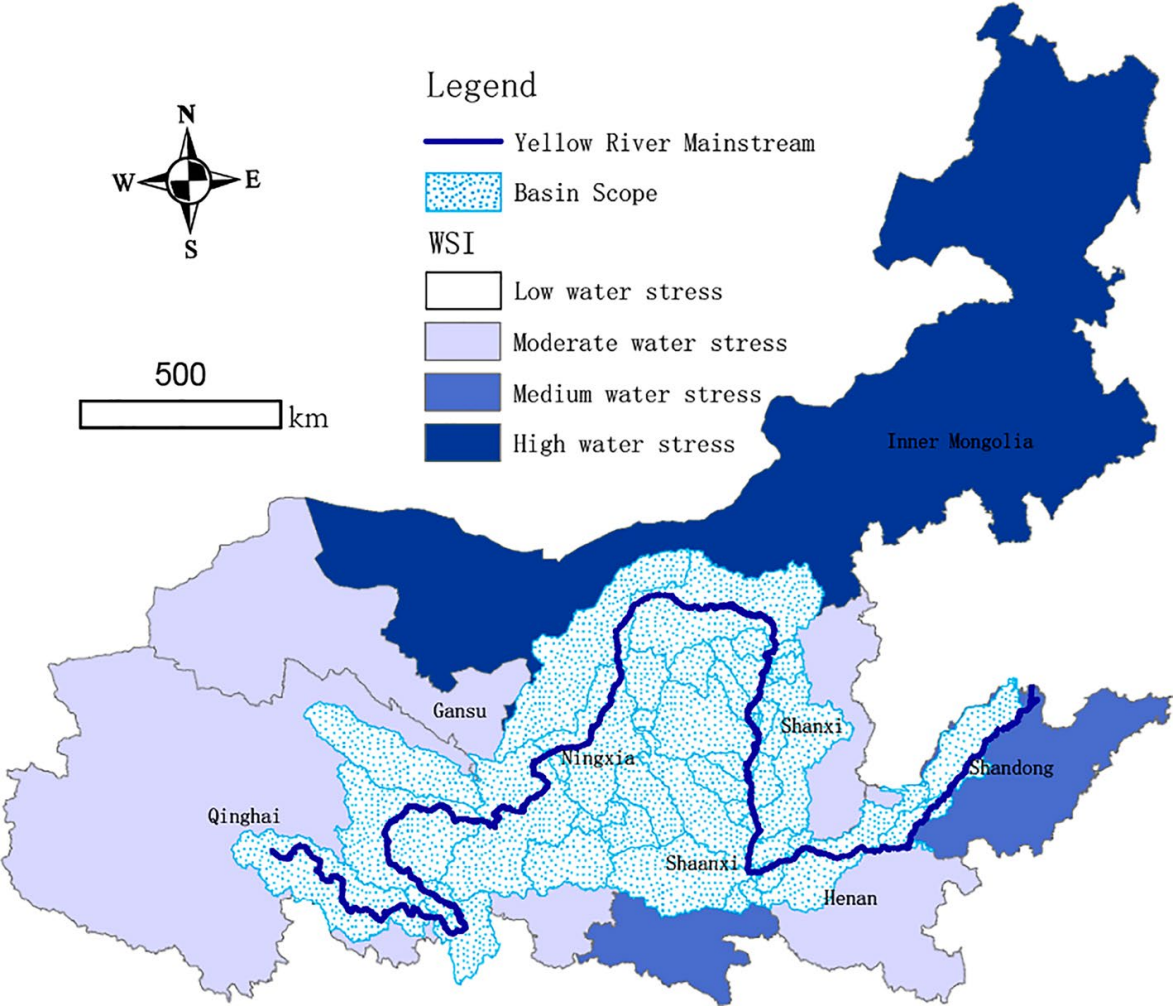
The geographical coverage of the Yellow River and the Water Scarcity Index (WSI) are mapped across different provinces.

As a typical water-scarce basin, water shortages impacting the socio-economic development constraints of the Yellow River region are increasingly prominent. First, the Yellow River water resource endowment is inherently insufficient. The per capita water of the basin is 905 m^3^ (approximately 1/3 of the national per capita water). The Yellow River contains 2.6% of the total water resources of the country, sustaining 12% of its population, 17% of its arable land area, and the water supply needs of more than 50 large and medium cities. Production and domestic water in the basin consume 5% of the already scarce ecological water. Second, the overall water resources development and utilization rate of the basin is too high, which is 70% and the rate of surface water is > 80%. Third, regional water resource utilization have exceeded the carrying capacity of the basin. The ratio of the amount of water withdrawn from each province in the Yellow River to the amount of water available for supply to that province reflects the water shortage tensions in the basin area, termed as the water stress index (WSI). The WSI and its classification criteria in the Yellow River region in 2017 are shown in Fig. 2 and Table A3, respectively. According to the WSI^49^, Qinghai, Gansu, Shanxi, and Henan are moderate water stress regions; Ningxia, Shanxi, and Shandong are medium water stress regions; and Inner Mongolia is a high water stress region.

### Data sources

We applied the latest published multiregional input–output (MRIO) table data of 42 sectors in 31 provinces of China in 2017 from the China Emission Accounts and Datasets (CEADs) database. The 42 sectors were collated into eight sectors (Appendix Table A3) according to the GB/T4754-2017 National Economic Sector Classification standard. Data on agricultural water use in each province were obtained from the 2017 China Water Resources Bulletin, and that on industrial water use were obtained from the China Statistical Yearbook on Environment 2018. Based on the input–output ratios of the “water production and supply industry” in different industries, the water consumption data of the mining, light, heavy, construction, and transportation industries were determined. Urban domestic water use in the China Statistical Yearbook consists of public (the service sector and construction industries) and residential water use. Therefore, the difference between urban domestic and residential water use was used to calculate the public water use. The average daily water consumption of each province in the Standard for Urban Residential Domestic Water Consumption (GB/T50331-2002)^50^ was multiplied by the urban water consuming population in the 2018 China Statistical Yearbook to obtain the urban residential daily water consumption. The details of the data sources are presented in Table 2.

**Table 2:** Data sources.

Data indicators and units	Data sources
MRIO by 42 departments in 31 provinces nationwide in 2017 (RMB million)	CEADs https://www.ceads.net/user/index.php?id=1090&lang=cn
Water withdrawal and water consumption by provinces in the Yellow River (billion m^3^)	Yellow River Water Resources Bulletin
Sectoral water consumption by province (billion m^3^)	Water Resources Statistical Yearbook and Water Resources Bulletin of each province
Water consumption in agriculture, industry, domestic and ecological environment by province (billion m^3^)	China Statistical Yearbook on Environment
Water consumption in the service sector (billion m^3^)	China Statistical Yearbook

## Results

### Virtual water flow at the sectoral level in the Yellow River Basin

#### Sectoral virtual water consumption intensities

Table 3 shows the total virtual water consumption intensity of each industrial sector in the Yellow River Basin from 2017, which includes the direct and indirect virtual water consumption intensities. The results reveal that the agricultural sector in the Yellow River Basin consumes the most water resources. To more comprehensively evaluate the water consumption degree during industrial development in the Yellow River Basin, the direct and indirect virtual water consumption intensity must also be analyzed at the sectoral level. The direct virtual water consumption intensity for agriculture and electricity accounted for 85.31% and 55.56% of the total virtual water consumption intensity, respectively. However, the total amount of water consumed by the light (2.01 m^3^/million yuan) and heavy (1.78 m^3^/million yuan) industries was larger than the amount of water they directly consume. However, these sectors consume numerous products from other industrial sectors as intermediate inputs simultaneously, which also consume a large amount of water resources during production. For example, the indirect virtual water consumption intensities of the mining, light, heavy, construction, transportation, and service industries accounted for 58.40%, 97.87%, 89.03%, 55.40%, and 79.01%, and 87.56% of the total virtual water consumption intensity, respectively. This suggests that most industrial sectors in the Yellow River are characterized by high indirect water consumption.

**Table 3:** Eight sectoral virtual water consumption intensity (m^3^/million yuan).

Sectors	Index		
	Direct virtual water consumption intensity	Indirect virtual water consumption intensity	Total virtual water consumption intensity
Agriculture	251.28	43.27	294.55
Mining	4.82	6.76	11.58
Light industry	2.01	92.53	94.54
Heavy industry	1.78	14.45	16.23
Production and supply of electricity, gas, and water	12.29	9.83	22.12
Construction industry	11.87	14.75	26.62
Transportation industry	1.51	5.70	7.21
Service industry	1.76	12.40	14.17

#### Magnitude of in-basin, out-of-basin, and export-embodied virtual water flows in the Yellow River Basin

**Figure 3:**
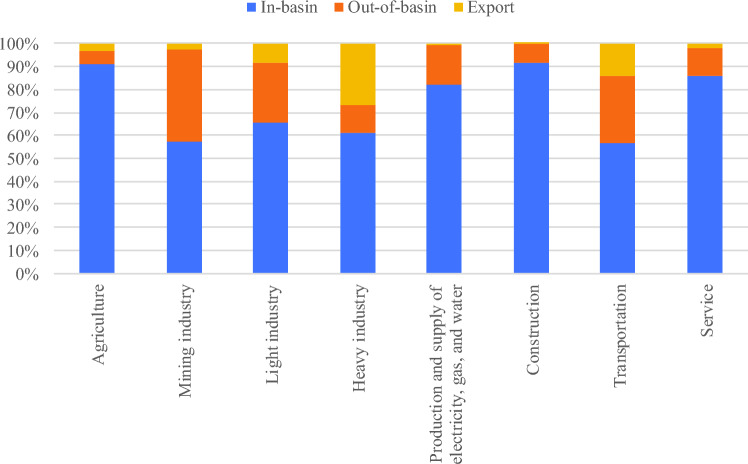
The figure illustrates the percentage distribution of virtual water flow structures across eight sectors in the Yellow River.

**Figure 4:**
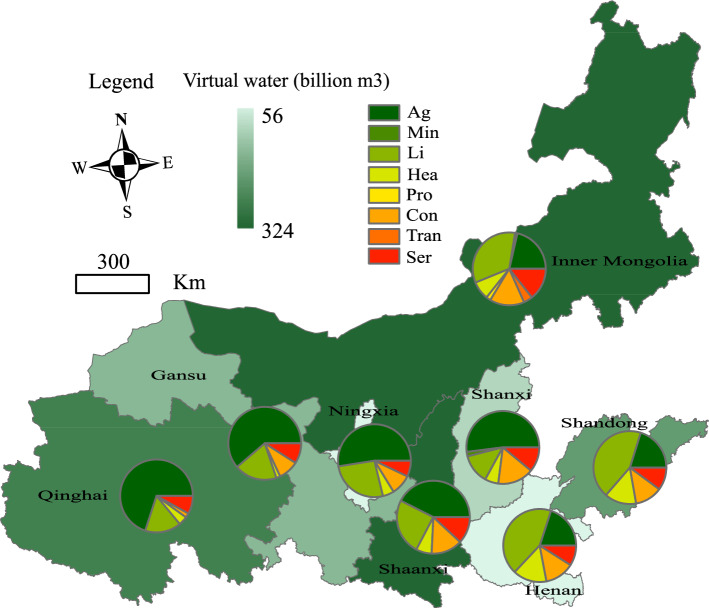
The virtual water flows of eight sectors in the Yellow River Basin (billion m^3^).

Figure A1 shows that the flow path of the basin virtual water system is classified as in-basin, out-of-basin, and export. The virtual water flow structure in terms of these flow categories for the eight sectors are shown in Fig. 3 and a map of the regional flow rate proportions is presented in Fig. 4. Overall, agriculture and light industries are the dominant sectors of virtual water trade in the Yellow River, at 13.58 billion m^3^ and 12.33 billion m^3^, respectively. The agricultural sector dominates virtual water trade in the basin, accounting for 90.75% of the total, whereas that out-of-basin accounted for only 5.70%. The Yellow River Basin, as the main grain producing area in China, has a large volume of regional and inter-regional agricultural virtual water trade^51^. The in-basin virtual water trade in the light industry accounts for 65.48% of the total, followed by virtual water exports accounting for 8.43%. The light industries in Shandong, Henan, and Inner Mongolia had the highest virtual water trades, at 4.51, 2.90, and 2.06 billion m^3^, respectively. Heavy industry, construction, and service sector virtual water trade volumes were relatively close, at 3.94, 4.68, and 4.22 billion m^3^, respectively. Heavy industry exports the largest proportion of virtual water trade volume (26.98%), followed by the virtual water trade volumes of the mining industry (98 million m^3^); electricity, gas, water production, and supply sectors (139 million m^3^); and the transportation industry (335 million m^3^). Moreover, the virtual out-of-basin water flow accounted for 24.75% of the total. Among these, the largest share of in-basin virtual water trade was in the mining (57.44%) and electricity, gas, and water production and supply sectors (82.41%). Finally, the out-of-basin and export virtual water trade volume of the transportation sector accounted for 43.38% of the total.

### Provincial virtual water trade in the Yellow River Basin

**Figure 5:**
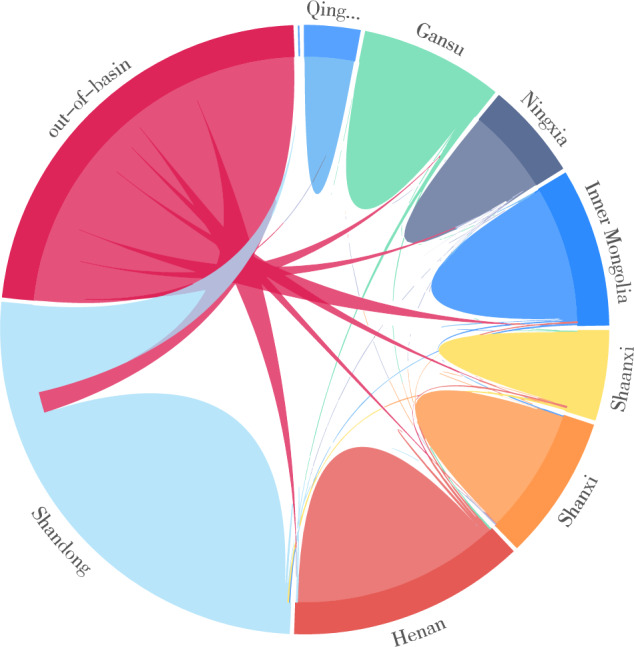
The virtual water flows among eight provinces of the Yellow River Basin in 2017 (billion m^3^).

Figure 5 shows the virtual water trade flows for the eight provinces of the Yellow River Basin in 2017, and the total amount of virtual water trade was 39.31 billion m^3^. Among these, 29.48 billion m^3^ (74.98%) of virtual water was transferred between the eight provinces in the basin, and 9.84 billion m^3^ (25.01%) of virtual water flowed out of the basin. Furthermore, 55.18% of virtual water was for domestic trade (5.43 billion m^3^) and 44.82% was for export (4.41 billion m^3^).

Two of the virtual water trade flow pattern characteristics were derived by calculating the inter-regional virtual water trade volume in the Yellow River Basin. First, the virtual water trade volume in the basin shows a gradually increasing trend from upstream to downstream and the volume of inter-regional virtual water trade decreases with the increase in regional distance. The virtual water trade volumes in Shandong and Henan (downstream of the basin) and Inner Mongolia (middle reaches) were larger, at 8.36, 5.43, and 3.62 billion m^3^, respectively. However, the virtual water trade volumes in Qinghai, Ningxia, and Shaanxi (upper reaches of the basin) were smaller, at 1.26, 2.19, and 2.05 billion m^3^, respectively. In addition to the inter-regional distance, regional natural geographical endowment, regional socio-economic development level, and inter-regional trade links are important factors affecting the virtual water trade volume^5^. Second, the inter-provincial trade has greatly reshaped water use patterns in the Yellow River Basin. The regional virtual water volume of Qinghai and Shanxi accounted for 91.04% and 83.68% of the total virtual water trade and the out-of-basin virtual water trades in Inner Mongolia, Shaanxi, Gansu, Ningxia, Henan, and Shandong were higher, with regional virtual water accounting for 61.48%, 66.82%, 69.19%, 69.78%, 72.38%, and 73.71% of the total.

### Excess and surplus water in the Yellow River Basin

**Figure 6:**
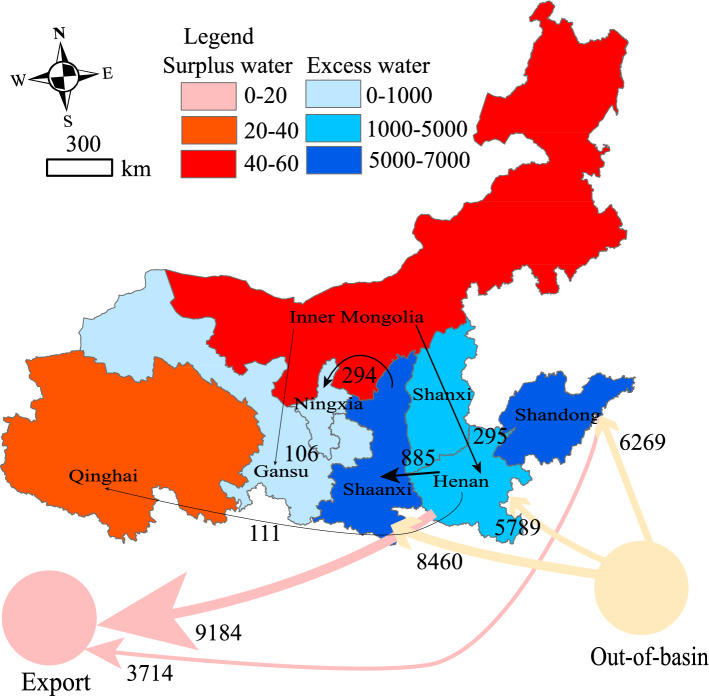
The virtual water in Yellow River Basin (billion m^3^).

The water resources available in the basin are insufficient to meet the total water demand. These regional excess water and surplus water values are shown in Table 4. The Yellow River is a virtual net water inflow area, importing 18.848 billion m^3^ of its water resources from outside the basin. Using Eq. (16), the net virtual water imports in the basin, total physical–virtual water, and the excess and residual water were calculated (Table 4) and mapped (Fig. 6). The total water resources of the Yellow River physical–virtual dual system in 2017 is 1742.627 billion m^3^, which exceeds the WPB of 115.533 billion m^3^, from the Three Red Lines (Table 1), and 873.32 billion m^3^, 40% of the total water resources. Notably, the physical water use was 51.66 billion m^3^, which is within the WPB.

**Table 4:** Excess water and surplus water in the Yellow River in 2017 (billion m^3^).

Province	Net Import virtual water (NIVM)	Physical water (PW)	Total physical–virtual water	SW (+) EW (−)	${W}_{r}^{*}$
Qinghai	18.303	1.583	19.886	3.686	− 0.113
Gansu	13.79	4.255	18.045	− 11.453	0.104
Ningxia	60.213	7.041	67.254	− 66.886	0.172
Inner Mongolia	5.695	9.61	15.305	6.175	− 1.149
Shaanxi	− 531.173	6.875	538.048	− 524.712	0.168
Shanxi	145.866	5.369	151.235	− 147.475	0.131
Henan	− 314.31	7.472	321.782	310.294	0.182
Shandong	601.617	9.455	611.072	− 604.336	0.231

Qinghai and Inner Mongolia are net virtual water inflow regions with surplus water; however, the net import of virtual water were less, at 18.303 and 5.695 billion m^3^, respectively, and the surplus water volumes were 3.686 and 6.175 billion m^3^, respectively. Overall, the virtual water volumes of Qinghai and Inner Mongolia accounted for 92.04% and 37.21% of the total physical–virtual water demand, respectively. The remaining provinces were excess water regions. Gansu, Ningxia, Shanxi, and Shandong were the virtual water net import regions, with virtual water net import values of 13.79, 60.213, 145.866, and 601.617 billion m^3^, respectively. The virtual water imports in these regions accounted for 76.42%, 89.53%, 96.45%, and 98.45% of the total regional physical–virtual water demand, respectively. This indicated that the excessive virtual water imports in these regions are the main reason for their regional water use exceeding the WPB. However, Shaanxi and Henan are net export regions of virtual water, with excess water volumes of 524.712 and 310.294 billion m^3^, respectively. The large net export of the virtual water volume in these regions (531.173 and 314.31 billion m^3^, respectively) was the primary cause of regional water use exceeding the WPB.

### Optimization results

While keeping the total available water resources in the Yellow River Basin at 51.916 billion m^3^ unchanged, the optimized and adjusted total amount of $\Delta {W}_{r}$, as indicated in Table 5, was 1.118 billion m^3^. The optimization results can be primarily classified into three categories. The first category includes Qinghai and Inner Mongolia, designated as surplus water regions, with recommended reductions of 0.113 and 1.005 billion m^3^ in their water allocation quotas, respectively. Inner Mongolia possesses the highest surplus water, totaling 6.175 billion m^3^, 1.67 times greater than Qinghai's. Furthermore, Inner Mongolia's net virtual water import stands at 5.695 billion m^3^, one-third of Qinghai's. These observations indicate that compared to Qinghai, Inner Mongolia is better equipped with the capability for water self-sufficiency and sustainable utilization. Therefore, a reduction in its water allocation quotas is justified. The second category involves the only two virtual water net-exporting provinces within the basin, namely Shaanxi and Henan. Their virtual water net exports account for 98.72% and 97.68% of the total physical–virtual water, respectively. This major factor contributes to the region exceeding the WPB for water usage. Therefore, Shaanxi and Henan, identified as excess water regions, should increase their water allocation quotas by 0.236 and 0.217 billion m^3^, respectively. The third category comprises regions such as Gansu, Ningxia, Shanxi, and Shandong, designated as excess water regions, which should increase their water allocation quotas by 0.098, 0.168, 0.236, 0.145, 0.217, and 0.254 billion m^3^, respectively.

**Table 5:** Optimization results of the water Allocation Scheme in 1987 (100 million m^3^).

Province	Qinghai	Sichuan	Gansu	Ningxia	Inner Mongolia	Shaanxi	Shanxi	Henan	Shandong	Hebei & Tianjin	Total
Water allocation scheme in 1987	14.1	0.4	30.4	40.0	58.6	38.0	43.1	55.4	70.0	20.0	370.0
2014 (South–North Water Diversion East-China Line Project in effect)	13.16	0.37	28.37	37.32	54.68	35.46	40.22	51.69	65.32	6.20	332.79
Water allocation in 2017	15.83	0.26	42.55	70.41	96.1	68.75	53.69	74.72	94.55	2.3	519.16
$\Delta {W}_{r}$	− 1.13	0	+ 0.98	+ 1.68	− 10.05	+ 2.36	+ 1.45	+ 2.17	+ 2.54	0	$\Delta$ 11.18
Optimized solutions	14.7	0.26	43.53	72.09	86.05	71.11	55.14	76.89	97.09	2.3	519.16

Data source: Comprehensive Plan of Yellow River, Yellow River Water Resources Bulletin.

Furthermore, the water transfer projects for physical water can be applied to optimize the water distribution scheme in the basin. In 2014, the South–North Water Transfer East-China Line Project achieved the first phase of water commissioning, considerably relieving water pressures in water-scarce areas such as Beijing, Tianjin, and Hebei. Therefore, the Yellow River Basin, as an excess water basin, should strictly control the scale of water transfer to areas outside the basin in the short term, for example, by gradually reducing the 2 billion m^3^ water allocation quota and the 230 million m^3^ water transfer quota of Tianjin and Hebei, respectively. The future allocation and utilization of water resources should be prioritized to meet the water supply needs of the basin. With the gradual completion of the South–North Water Diversion Project in the future, the water supply from the upper reaches of the Yellow River would inevitably increase. When increasing the amount of water allocated to the entire Yellow River, the proportion of water allocated to the middle and lower reaches of the basin should be increased accordingly. The West Line Project is expected to deploy 17 billion m^3^ of water resources to the upstream regions, which can alleviate water shortage in the upper and middle reaches of the Yellow River in 2050.

## Discussion and conclusion

We calculated the virtual water trade volume between regions in the basin via an input–output model; developed the optimal allocation model of the basin physical–virtual dual water system (to optimize the Yellow River 1987 water allocation scheme for 2017), which described the internal structure of the water system; obtained the overall WPB value of the water system; and designed different water allocation schemes for the excess and surplus water regions.

### Subsystem of basin physical–virtual water

The size of the physical water system stock and the trade flows of the virtual water system in the Yellow River are mismatched. Inter-regional virtual water trade has significantly reshaped water use in the basin^52^. Considering total water consumption, the regional physical system had less water and insufficient number of water resources are available in the basin. Virtual water consumption accounted for > 90% of the total; therefore, the structure and efficiency of water resource utilization should be improved considering the sector structure.

Further, agriculture and light industries were the primary regions of virtual water inter-regional trade. It is consistent with the study by Zhang et al.^53^, Li et al.^54^, and Zhang et al.^55^, who showed that the main virtual water sectors and the Yellow River will still be under pressure of water usage in future. The complete water use coefficients of the two sectors in 2017 were 294.55 and 94.54 m^3^/million yuan, respectively. The mining, heavy, and transportation industrial sectors accounted for a large share of virtual water exported to areas outside the basin (42.56%, 38.98%, and 43.38% respectively), which was the dominant cause of regional water shortage. The Yellow River Basin has abundant coal resources and the mining and heavy industrial sectors developed mainly based on these resources are important sectors of the energy output in China^56^. These regions must actively promote the improvement of renewable and energy storage technologies to accelerate the transformation of traditional energy sectors, such as electricity and gas. Simultaneously, the proportion of renewable energy consumption, such as wind and solar energy, will be increased to ultimately achieve two-way water saving and carbon reduction benefits.

The dominant reason for the regional occurrence of water use exceeding the WPB in the Gansu, Ningxia, Shanxi, and Shandong provinces (net virtual water import regions) is their excess virtual water imports. Qinghai and Inner Mongolia, with relatively abundant water resources in the basin, are net import regions in the high water intensity sector. The water-poor regions in the basin, such as Shandong and Henan, are the main grain producing regions with high water-consuming agricultural industries that dominate the industrial structure and supply high water intensity products to other water-rich regions in the basin. This virtual water system cycle further exacerbates the degree of overall water system imbalance in the basin regions.

Therefore, the virtual water trade can be considered a policy tool to address the industrial and regional allocation mismatch of the physical–virtual water system in the Yellow River Basin^57^. For example, the regional water shortage may be solved by exporting efficient and low-consumption water products and importing water products and services that are not available locally. The agricultural and light industrial sectors with large virtual water content provide essential products and services for coastal-basin residents and are characterized by low elasticity demand. For the dominant virtual water trade sectors in the basin, improving water use efficiency in the agricultural sector and adopting water conservation policies in the light industrial sector are crucial. Reducing energy product supplies with high energy and water consumption and increasing the proportion of imported virtual water in the high water-consumption density sectors can effectively save water resources in the basin.

### Basin water planetary boundary and water allocation optimization scheme

Water resource allocation in the Yellow River must be adapted to regional WPB thresholds. By identifying the basin WPBs and establishing excess and surplus water indicators, this study calculated the excess and surplus water use of the regional water system. According to the “replenishing and reducing excess water” principle, the water distribution quotas of the surplus water regions in Qinghai and Inner Mongolia were reduced, which is consistent with the results of Ye et al.^28^ and Zhao et al.^58^. There were several Pareto solutions in the optimization model, which could be regarded as ideal points due to the higher economic benefit and lower water consumption compared to other studies that exclusively focus on the physical water system in the Yellow River Basin water resource optimization^59,60^. Increasing the water allocation quota for the remaining excess water regions is also important to optimize water allocation. The excess and surplus water indicators can help alleviate regional water shortage caused by commodity trade in high water-consuming sectors. The results showed that optimal allocation integrated with virtual water trade could rationalize the export–import of products at the basin scale, which is consistent with the results of Chen et al.^32^. However, the optimal allocation model of the basin physical–virtual dual water system developed in this study requires further exploration.

First, the optimization scheme necessitates that ecological water quotas are not reduced. The natural water system is critical to the stable operation of the basin-wide water system, and a shortage of ecological water can hinder the stable operation of the natural basin water system. According to the “three red lines” policy, to optimize the basin water distribution scheme, the safe operation of the natural water system in the basin must be ensured. Specifically, to prevent degradation of the water ecology and environment of the basin, the amount of water reserved for ecological use in the original river cannot be reduced, as it is unified by government departments. Second, if the optimized water allocation scheme obtained based on the model established in this study cannot meet the regional water demand, the next step requires the consideration of establishing a physical water transfer project. Inter-basin water transfer engineering is a common strategy to alleviate water shortage in the physical–virtual water subsystem. However, this approach can only be used as a supplementary regulation tool to solve regional water shortage problems. For example, the South–North Water Transfer Project has greatly alleviated water shortage in the downstream regions of the Yellow River Basin, such as Henan, Hebei, Tianjin, and Shandong. Additionally, inter-basin water transfer projects such as the ecological replenishment of the Yongding River into the Yellow River have also indirectly influenced the design of the Yellow River water allocation scheme.

### Limitations and future work

The Yellow River Basin is the first basin in China to implement unified basin-wide water allocation and scheduling. It has a large area with complex water-system relationships, and many interested parties and sectors are involved with no precedent experience to follow. Therefore, studying water resource optimization schemes in the basin is preceded by theory^61^, and requires an optimization process of constantly identifying problems, summarizing experience, and continuously implementing practical adjustments. Our study has some limitations, which represent crucial future research directions.

First, this optimal water allocation scheme is a dynamic adjustment process. For a long time-series, the incoming water of the physical water system is spatiotemporally abundant but depleting. Therefore, the physical water allocation optimization principle requires adjustment according to the basin-specific annual water abundance. This method can be extended using the “increase of abundance and decrease of drought” principle from the 1987 water allocation scheme. Second, detailed data on water availability and consumption in China are limited; therefore, future studies are required to develop more comprehensive accounting methods and systems for water management in China. Finally, the optimization model developed in this study can be utilized to guide ecological compensation and cross-basin water resource allocation. Ecological compensation is an important tool for regulating economic and ecological interests across basin regions. The ecological compensation mechanism should be established and improved from the perspective of the basin physical–virtual multi-subsystem nature^62^. Based on the market water price, the Chinese government should further improve the national water resource allocation and ecological compensation plan with virtual water as the main means.

### Supplementary Information

Supplementary Information.

## Data Availability

The datasets used and analysed during the current study available from the corresponding author on reasonable request.
