# Supplementary material for: Optimal water resource allocation considering virtual water trade in the Yellow River Basin
Source: Sci Rep. 2024 Jan 2;14:79. doi: 10.1038/s41598-023-50319-6 (PMC10761993; doi:10.1038/s41598-023-50319-6)
Supplement: Supplementary file 1 — Supplementary Information. [file 41598_2023_50319_MOESM1_ESM.docx]

**Table A1.** Standard structure of water resources input-output table (economic flow table) in the Yellow River

| Output/Input | | | Intermediate Use | | | | | | | | | | | | | | | | | | | Total Final Use | Export | Gross Output |  |
| --- | --- | --- | --- | --- | --- | --- | --- | --- | --- | --- | --- | --- | --- | --- | --- | --- | --- | --- | --- | --- | --- | --- | --- | --- | --- |
|  |  |  | Qinghai | | | Gansu | | Ningxia | | Inner Mongolia | | Shaanxi | | Shanxi | | Henan | | Shandong | | | Field | F | E | X |  |
|  |  |  | Sector1  ...  Sector8 | | |  | |  | |  | |  | |  | |  | |  | | |  |  |  |  |  |
| Intermediate Input | Qinghai | Sector1  ...  Sector8 |  |  |  | |  | |  | |  | |  | |  | |  | |  |  | |  |  |  | |
|  | Gansu |  |  |  |  | |  | |  | |  | |  | |  | |  | |  |  | |  |  |  | |
|  | Ningxia |  |  |  |  | |  | |  | |  | |  | |  | |  | |  |  | |  |  |  | |
|  | Inner Mongolia |  |  |  |  | |  | |  | | Z | |  | |  | |  | |  |  | |  |  |  | |
|  | Shaanxi |  |  |  |  | |  | |  | |  | |  | |  | |  | |  |  | |  |  |  | |
|  | Shanxi |  |  |  |  | |  | |  | |  | |  | |  | |  | |  |  | |  |  |  | |
|  | Henan |  |  |  |  | |  | |  | |  | |  | |  | |  | |  |  | |  |  |  | |
|  | Shandong |  |  |  |  | |  | |  | |  | |  | |  | |  | |  |  | |  |  |  | |
|  | Field |  |  |  |  | |  | |  | |  | |  | |  | |  | |  |  | |  |  |  | |
| Import | | |  |  |  | |  | |  | | IM | |  | |  | |  | |  |  | |  |  |  | |
| Total Value Added | | |  |  |  | |  | |  | | V | |  | |  | |  | |  |  | |  |  |  | |
| Total Input | | |  |  |  | |  | |  | | X | |  | |  | |  | |  |  | |  |  |  | |
| Water Consumption | | |  |  |  | |  | |  | | W | |  | |  | |  | |  |  | |  |  |  | |

**Table A2.** 2017 China Multi-Regional Input-Output Tables 42 Sectors Consolidated 8 Sectors

| 8 Sectors | Number | 42 Sectors |
| --- | --- | --- |
| Agriculture | 1 | Agriculture, Forestry, Animal Husbandry, and Fishery |
| Mining industry | 2 | Mining and washing of coal |
|  | 3 | Extraction of petroleum and natural gas |
|  | 4 | Mining and processing of metal ores |
|  | 5 | Mining and processing of nonmetal and other ores |
| Light industry | 6 | Food and tobacco processing |
|  | 7 | Textile industry |
|  | 8 | Manufacture of leather, fur, feather, and related products |
|  | 9 | Processing of timber and furniture |
|  | 10 | Manufacture of paper, printing, and articles for culture, education, and sports activity |
| Heavy industry | 11 | Processing of petroleum, coking, processing of nuclear fuel |
|  | 12 | Manufacture of chemical products |
|  | 13 | Manuf. of non-metallic mineral products |
|  | 14 | Smelting and processing of metals |
|  | 15 | Manufacture of metal products |
|  | 16 | Manufacture of general-purpose machinery |
|  | 17 | Manufacture of special-purpose machinery |
|  | 18 | Manufacture of transport equipment |
|  | 19 | Manufacture of electrical machinery and equipment |
|  | 20 | Manufacture of communication equipment, computers, and other electronic equipment |
|  | 21 | Manufacture of measuring instruments |
|  | 22 | Other manufacturing and waste resources |
|  | 23 | Repair of metal products, machinery, and equipment |
| Production and supply of electricity, gas, and water | 24 | Production and distribution of electric power and heat power |
|  | 25 | Production and distribution of gas |
|  | 26 | Production and distribution of tap water |
| Construction | 27 | Construction |
| Transportation | 29 | Transport, storage, and postal services |
| Service | 28 | Wholesale and retail trades |
|  | 30 | Accommodation and catering |
|  | 31 | Information transfer, software, and information technology services |
|  | 32 | Finance |
|  | 33 | Real estate |
|  | 34 | Leasing and commercial services |
|  | 35 | Scientific research |
|  | 36 | Polytechnic services |
|  | 37 | Administration of water, environment, and public facilities |
|  | 38 | Resident, repair, and other services |
|  | 39 | Education |
|  | 40 | Health care and social work |
|  | 41 | Culture, sports, and entertainment |
|  | 42 | Public administration, social insurance, and social organizations |

**Table A3.** WSI classification criteria

| WSI Index | [0,0.1] | [0.1,0.2] | [0.2,0.4] | [0.4,1] |
| --- | --- | --- | --- | --- |
| Classification | Low water stress | Moderate water stress | Medium water stress | High water stress |

**Table A4.** Total water resources of the physical-virtual dual system of Yellow River in 2017 (billion m^3^)

| Province | Local outflow virtual water | The local inflow of virtual water | NIVW(Net import virtual water) | Physical water | Total physical-virtual water |
| --- | --- | --- | --- | --- | --- |
| Qinghai | 5473.41 | 5656.44 | 183.03 | 15.83 | 198.86 |
| Gansu | 11063.13 | 11201.03 | 137.90 | 42.55 | 180.45 |
| Ningxia | 5296.86 | 5898.99 | 602.13 | 70.41 | 672.54 |
| Inner Mongolia | 19493.18 | 19550.13 | 56.95 | 96.10 | 153.05 |
| Shaanxi | 26846.29 | 21534.55 | -5311.73 | 68.75 | 5380.48 |
| Shanxi | 22732.25 | 24190.91 | 1458.66 | 53.69 | 1512.35 |
| Henan | 82560.21 | 79417.11 | -3143.10 | 74.72 | 3217.82 |
| Shandong | 14555.55 | 20571.72 | 6016.17 | 94.55 | 6110.72 |

**Fig. A1.** The flow path of the virtual water system in the Yellow River basin

**Fig. A2.** Genetic algorithm solver
